# Operationalized psychodynamic diagnosis as an instrument to transfer psychodynamic constructs into neuroscience

**DOI:** 10.3389/fnhum.2013.00718

**Published:** 2013-10-25

**Authors:** Henrik Kessler, Michael Stasch, Manfred Cierpka

**Affiliations:** ^1^Department of Psychosomatic Medicine and Psychotherapy, LWL-University, Ruhr-University BochumGermany; ^2^Institute for Cooperation Research and Family Therapy, University of HeidelbergHeidelberg, Germany

**Keywords:** neuroscience, psychoanalysis, neuropsychoanalysis, operationalized psychodynamic diagnosis, individualization, fMRI

## Abstract

This theoretical article makes a contribution to the field of “psychoanalytically informed neuroscience”. First, central characteristics of psychoanalysis and neuroscience are briefly described leading into three epistemic dichotomies. Neuroscience versus psychoanalysis display almost opposing methodological approaches (reduction vs. expansion), test quality emphases (reliability vs. validity) and meaning of results (correlation vs. explanation). The critical point is to reach an intermediate level: in neuroscience an adequate position integrating both aspects—objective and subjective—of dual-aspect monism, and in psychoanalysis the appropriate level for the scientific investigation of its central concepts. As a suggestion to reach that level in both fields the system of Operationalized Psychodynamic Diagnosis (OPD; OPD Task Force, 2008) is presented. Combining aspects of both fields areas, expansion and reduction as well as reliability and validity, OPD could be a fruitful tool to transfer psychodynamic constructs into neuroscience. The article closes with a short description of recent applications of OPD in neuroscience.

## Introduction

Despite many efforts to bring psychoanalysis and neuroscience together, we propose that there exists a profound gap between the two approaches. Hence, the intention of this theoretical article is, first, to describe those core differences and provide possible explanations for them and, second, to present the system of Operationalized Psychodynamic Diagnosis (OPD; OPD Task Force, 2008) as an instrument to bridge both sides. Although there exists a long (and eventful) relationship between psychoanalysis and different forms of neurosciences, the term “neuropsychoanalysis” itself was only coined in 1999 with the title of the respective journal. In an attempt to bring psychoanalysis and neuroscience together, pioneers in the field, Mark Solms and Oliver Turnbull, describe two scientific areas that fall under their broad conception of neuropsychoanalysis (Solms and Turnbull, 2011). First, they refer to the direct psychoanalytic investigation of neurological phenomena. This was the starting point of their work in the field using mainly patients with focal brain damage (Kaplan-Solms and Solms, 2000). Second, they also include psychoanalytically informed neuroscience, where hypotheses derived broadly from psychoanalysis are tested with neuroscientific methods. It is only the second aspect of neuropsychoanalysis that will be the subject of this article. On the theoretical and empirical side, many endeavors bringing both fields together could be observed which were mainly propelled by methodological and conceptual progresses achieved in neuroscience. Those endeavors are covered in the review by Sauvagnat and colleagues (Sauvagnat et al., 2010). Looking at institutions and structures, the founding of the “International Neuropsychoanalysis Society” and its respective journal *Neuropsychoanalysis* was a major step towards “building bridges between psychoanalysis, neuroscience, psychology and psychiatry” (full header on the society’s website). Importantly, the society members and editorial staff of the journal include prominent scientists from both fields. This special issue on neuropsychoanalysis in *Frontiers in Human Neuroscience* is another venture assisting the “genuine dialogue between biology and psychoanalysis” (Kandel, 1999).

So far, there seem to be co-operations and exchanges between the two fields. Nevertheless, we still notice a strong reductionism in neuroscience and, more importantly, biological psychiatry. Psychoanalytic concepts seem to be virtually absent in major publications reporting empirical contributions in biological psychiatry. It is important, at this point, to introduce the differentiation between methodological and ontological reductionism. Only the latter, the idea that mental disorders *are* merely wrongly wired neurons or imbalances in certain transmitters and that it is of no relevance to consider psychological aspects (ontological reductionism) is questioned here. The *methodological* reductionism is a necessary prerequisite of neuroscience, stressing the importance to reduce complex phenomena into components that can be studied with neuroscientific methods. This form of reductionism is further described in the next section and actually forms one aspect where both, neuroscience and psychoanalysis, could be connected. On the psychoanalytical side there are still articles questioning the value of neuroscience to psychoanalysis in principle (Blass and Carmeli, 2007). Harder to capture within the scope of a scientific article though, but an important part of our reality, is the fact that there are psychoanalysts questioning the approaches, methods and results of existing neuropsychoanalytic empirical work in seminars and discussion forums. There seems to be a profound scepticism towards attempts to bridge the two areas.

Our critical point is that the gap is not merely a problem of different scientific languages. From our point of view, the differences between neuroscience and psychoanalysis go deeper and can be dichotomized into almost opposing methodological approaches (reduction vs. expansion), test quality emphases (reliability vs. validity) and meaning of results (correlation vs. explanation). This will be embellished in the next section. Two intersections follow that are concerned with the problem to reach an intermediate level in both, psychoanalysis and neuroscience. Afterwards, OPD as a system will be presented, and the last section explains how OPD could help to transfer psychodynamic constructs into neuroscience, propelling empirical research in the field that Solms and Turnbull (2011) named “psychoanalytically informed neuroscience”.

## Characteristics of psychoanalysis and neuroscience

When considering differences between psychoanalysis and neuroscience one has to be aware of the divergent aims of both approaches. Whereas psychoanalysis was developed as a genuinely therapeutical technique to help the individual patient regain mental health, neuroscience wants to uncover the neural foundations of mental processes on an experimentally controlled level intentionally abstracting from the individual. From those divergent aims, different characteristics can easily be derived.

In psychoanalysis, the starting point is a clinical problem, i.e., a patient seeking for help from the psychoanalyst because he or she suffers from some form of mental disorder. To better understand the patient, the psychoanalyst eventually gathers information “surrounding” the problem: biographical background, recent circumstances of living and at work, situations triggering the problem, interpersonal relations, wishes, hopes, fears, complaints, dreams, and many more. That information is obtained by listening to the patient with “evenly suspended attention” (Freud, 1914) and sometimes asking direct questions. In other words: the initial clinical problem (“I feel depressed”) is *expanded* into various branches of the patient’s life and environment, in width and depth, to obtain a more comprehensive picture. All of this time-consuming process has the goal to, plainly spoken, “find out what really bothers the patient”. That is, what are the factors that cause and eventually maintain the patient’s clinical problem? Those factors could be idiosyncratic ways of interpersonal relations, certain psychodynamic conflicts or structural deficits. All of those factors are mainly rooted in the patient’s biographical experiences (Person et al., 2005), the emotional and behavioral reactions towards those experiences, and finally the mental representation of this idiosyncratic experience and behavior. In psychoanalysis the idea of psychic determinism (Brenner, 1974) is of central importance: the above mentioned factors are believed to play a genuinely causal role in the development of the individual problem. This leads to two epistemic consequences. First, gathering information and factors can only be fruitful, if it deals with themes that actually apply to the individual, that are “truly his issues”. Hence, on the level of test quality (although a psychoanalyst would hardly use the term regarding his practice), *validity* is of key importance. Second, causal inferences from psychological factors to clinical problems allow for an *explanation* of the latter. Dysfunctional relationship patterns with the parents could, for instance, explain the patient’s problems in recent relationships leading to social isolation and depressed mood. *Expansion, validity* and *explanation* are thus three epistemic hallmarks in the practice of psychoanalysis.

As for neuroscience, the need for experimental control and abstraction from the individual call for almost opposing characteristics. When designing an experiment dealing with a certain psychological process, the inherent complexity of that very process has to be strictly controlled in order to achieve any meaningful results at all. One typical way of control is the *reduction* of a complex process into simpler components, which themselves are subject to the empirical investigation. Only relatively simple (sub-) components can be varied systematically in order to find regularities and differences in their characteristics. Furthermore, systematic experiments should be replicable. Investigating group effects for a certain psychological process and abstracting from individuals, different labs should principally obtain the same results using the same experiment. Hence, *reliability* is the central issue from a test quality perspective. Finally, due to well-investigated methodological constraints (e.g. Logothetis, 2008), results obtained with the most common neuroscientific methods (e.g., neuroimaging) reflect merely psycho-physiological co-activation. That is, the activity of certain brain areas that timely co-occurs with an observed psychological process is typically interpreted as the neural *correlate* of this very process. Thanks to the caution of serious neuroscientists, causality would not be inferred from this activity. Consequently, though, the measured brain activity cannot explain the psychological process. *Reduction, reliability* and *correlation* are hence the neuroscience counterparts of the above mentioned aspects in psychoanalysis.

## Intersection I: Reaching the intermediate level in neuroscience

We dichotomized psychoanalysis and neuroscience in the previous section, but in fact profound differences in perspective exist even within each area. This shall be outlined briefly. As for neuroscience (and adjacent disciplines), a core—and very old—problem remains the question how brain and mind relate, that is, how can we bring together two entities that are apparently so different. Many endeavors in philosophy, psychology and other disciplines have been made to tackle this issue, but in the growing field of neuropsychoanalysis a position prevails that can be described as dual-aspect monism or perspectivism (see Solms, 1997 and Solms and Turnbull, 2002 for a detailed description and an overview of the debate). The central idea is that mind and brain are the same (monism), but we perceive them from two different perspectives (dual-aspect). As neuroscientists we focus on the physiological and anatomical aspect of the mind/brain from an “objective” perspective, and as human beings we perceive it from the inside in a “subjective” way. Regardless of how we see it, mind/brain remains one entity. Sigmund Freud also adopted this “intermediate” position and provided us with the technique of free association as a means to explore the inner, subjective, aspect of mind/brain. The two premises of dual-aspect monism—mind and brain are one that we can perceive from two perspectives—actually form the very foundation of the neuropsychoanalytic venture. The “monism” part is implicitly included when researchers like Eric Kandel state in an influential article bridging psychiatry and biology: “Insofar as our words produce changes in our patient’s mind, it is likely that these psychotherapeutic interventions produce changes in the patient’s brain” (Kandel, 1998, p. 466). In this article, Kandel understands interactions between brain, mind and behavior on a broad level: behavior and social factors exert actions on the brain by feeding back upon it to modify gene-expression and thus nervous functioning. It is hence the belief that psychosocial experiences actually have an impact on the physical brain, that relationship patterns leave their traces in neural networks that fuels the research in neuropsychoanalysis. The true art is, of course, to reach that intermediate level in neuroscience where both perspectives—the objective and subjective—can best be integrated. The application of OPD in neuroscientific research (see last section) is an attempt to reach that level.

## Intersection II: Reaching the intermediate level in psychoanalysis

Psychoanalysis itself is facing the difficulty to reach an intermediate level appropriate for the scientific investigation of its central concepts. Hence, a brief introduction into the problem of operationalization in psychoanalysis is provided before describing the system of OPD itself.

Attempts to operationalize psychoanalytic concepts are inevitably encountered with challenging difficulties. The central task of the operationalization of a construct must be to establish a link between the levels of theory and observation. Research operationalizations are primarily geared towards the logic of experimental design, and in this way substantially influence the translation of the original theoretical term into an operational term. For this, it is necessary to explicate and specify the relevant constructs in order for them to be translated into research operations. This step is difficult in psychoanalysis with its complex constructs like the unconscious, processes of repression, affects, or transferences which are not directly observable but must be inferred from their “derivatives”. Therefore endeavors of operationalization of psychoanalytic concepts must reach an intermediate level, which may allow a gain in clarity and unequivocalness, without at the same time removing the concept too far away from its dynamic content. To emphasize, the goal of such a procedure would be to gain as much freedom from contradiction as possible while preserving as much dynamic content as possible.

## Operationalized Psychodynamic Diagnosis (OPD)

As for the “standard” assessment of mental disorders the “Diagnostic and Statistical Manual” (DSM-IV) of the American Psychiatric Association (APA, 1994) and the “International Classification of Mental and Behavioral Disorders” (ICD-10) of the World Health Organization (WHO, 1992) have attained wide usage. Psychodynamic psychotherapists (and others) regret, however, the lack of relevance of the phenomenological and symptom-centered diagnoses of ICD and DSM when seeking possible explanations of clinical problems. On the other side, there was a growing dissatisfaction with the divergence of psychoanalytic theory: different groups vary in terminology and develop their own (sub-) concepts, rendering communication between therapists difficult. Those two problems were the starting point for the creation of the OPD Task Force in 1990 in Germany. The goal was to broaden the ICD and DSM classifications to include fundamental psychodynamic dimensions, and at the same time to remain aspects of reliability and terminological precision apparent in ICD and DSM. In reference to the previous section, OPD is an attempt to reach the intermediate level.

The OPD system is based on four psychodynamically relevant diagnostic axes with appropriate categories to complement ICD classification (fifth axis): axis I (experience of illness and prerequisites for treatment), axis II (interpersonal relations), axis III (psychodynamic conflicts), axis IV (psychological structure) and axis V (syndromal diagnosis according to ICD-10).

In practice, for a 1–2 h patient examination, which is still an open psychodynamic interview in nature, OPD provides flexible interview guidelines to ensure the relevant information is obtained. Details can be found in the recent OPD manual (OPD Task Force, 2008). The three axes most relevant for the psychodynamic approach will be described briefly.

As for axis II (interpersonal relations), mental disorders are conceived as “relationship disorders”. In almost all schools of psychotherapy, automated and maladaptive interpersonal behavior patterns are considered to be an essential factor influencing mental disorders. Along with symptomatic complaints, problems relating to interaction with others are often the most important factor to be addressed at the outset of psychotherapy. With time, through the “depositing” of relational experiences, mental representations develop alongside the life story, and these are confirmed or modified by our experiences in interpersonal relationships with others (Anchin and Kiesler, 1982). The basic structure of the OPD relationship axis depicts the circular or transactional character of human interaction (interchange of subjective experience and response to the environment). A framework was developed which encapsulates subjective experience concerning self and others on the initial level. On a second level it is possible to represent the experience of this other person (significant other, interviewer): how is the patient experienced by his objects or the interviewer and which impulses does he generate in them? Items of the OPD relationship axis help to define the variety of behaviors seen in relationships.

Axis III assesses psychodynamic conflicts searching for common motives in central life areas such as relationship to partner, family of origin, profession, ownership, behavior in groups and illness experience. OPD distinguishes the following seven conflicts: (1) Dependence versus autonomy; (2) Submission versus control; (3) Desire for care vs. autarchy; (4) Conflicts of self-value; (5) Guilt conflicts; (6) Oedipal conflicts; (7) Identity conflicts. Those conflicts are operationalized thoroughly in the manual, providing a terminologically clear description of the conflict characteristics, its typical core affect, transference, counter-transference and implications for various aspects of the patient’s life.

Finally, the fourth axis is concerned with the psychological structure of the patient. OPD differentiates four levels of structure (good integrated, moderately integrated, low integrated, disintegrated). Good integration means that an autonomous self possesses a mental internal space in which mental conflicts can be carried out. Moderate integration implies lower availability of regulating function and a weaker differentiation of mental substructures. With low integration the mental inner space and substructures are less developed, thus conflicts are barely mentally worked out, but are mainly worked out in the interpersonal sphere. Disintegration is characterized by fragmentation and psychotic restitution of the structure. Operationalization of structure is based on four structural dimensions with a self-related and an object-related subdomain each: (1) Perception; (2) Regulation; (3) Communication; (4) Bonding. Again, those structural levels are operationalized extensively with clear descriptions and patient examples to illustrate specific structural deficits.

Concluding this section, OPD combines the best of both approaches: the inclusion of an expanded view apparent in psychoanalysis and the systematic reduction helpful in all experimental sciences (e.g. neuroscience). Due to the consideration of psychoanalytical constructs that are well-grounded in clinical experience, validity of the OPD is sufficient, and because of the systematic operationalization of assessment, reliability is constantly high in empirical investigations (Cierpka et al., 2007).

## The transfer of psychodynamic constructs into neuroscience

As a starting point, we do believe that psychoanalysis and neuroscience could substantially benefit from each other in the field of “psychoanalytically informed neuroscience” (Solms and Turnbull, 2011). The key question regards an adequate methodological approach to bring the two together. Being the main message of this article, we want to propose OPD as an instrument to transfer psychodynamic constructs into neuroscience. Advanced neuroscience methods offer superior experimental control and fine-grained analyses of brain activation and connectivity. This rigorous method is the bottom line for any scientific investigation of complex mental phenomena. To avoid the shortcomings of reductionism, that necessarily come with experimental control, an expansive tapping into real-world complexity should be tried, however. One important way to achieve this is via individualization of experiments, as has been proposed before (Kessler et al., 2011b). Only if the experiment touches the mentally represented themes that are of individual relevance to each subject, results could have validity and meaning in a deeper sense. But how can we gather individualized information in a systematic way that is compatible with experimental control? This is where OPD offers the system and practical tools for the task at hand. First, real-life complexity of actual patients is *reduced* into simpler components in a methodologically rigorous and transparent way as reflected in the axes and the differentiation between items within a given axis. The good operationalization of the OPD manual fosters *reliability*, and the richness (*expansion*) of the material obtained in an individual interview provides the basis for *validity.* Second, the components assessed with OPD can be translated into experimental stimuli with relative ease (see below). Brain reactions to those stimuli could be interpreted on a better foundation due to the individual and valid nature of the stimuli. This clinically-grounded *explanation* of brain activity must not, however, be confused with causality in a strict sense. Since there are always alternative directions to consider brain activity (see Lewis, 2000 and Kessler et al., 2011b for details) the quest for causality has to remain an open issue.

In the remainder of this section we want to give recent examples of how OPD is used in neuroscience. With space constraints and the focus of this article on the theoretical aspects, this part is kept short and serves mainly for illustratory purposes. Details can be found in the respective publications. The first study in this vein investigated patients with chronic depression undergoing psychoanalysis (Kessler et al., 2011a). An OPD interview was conducted with each patient to extract his or her major dysfunctional repetitive relation leading to depression or maintaining it. This pattern was translated into four essential sentences presented in the fMRI scanner (block design, 30 s for all four sentences). An example set would be: “You wish to be accepted by others. Therefore you do a lot for them. That is often too close for them, so they retreat. Then you feel empty and lonely”. Patients exhibited relatively strong activation in limbic and subcortical areas (e.g., amygdala, basal ganglia) reflecting possible emotion processing. The critical issue here is that brain responses to those stimuli can be interpreted on a clinical ground due to the individualized nature of the stimuli. In another study, OPD was used to derive sentences that capture the essence of typical psychodynamic conflicts (Schmeing et al., 2013). Those sentences served as stimuli in an fMRI experiment investigating free associations to potentially conflictual situations in healthy participants. Two types of conflict were used for the generation of “OPD” sentences: autonomy/dependency (e.g., “I cannot say “No” if someone else is asking me for help”), and self-esteem–conflict (e.g., “I often estimate myself as little competent”). Again, with the stimuli themselves being rooted in OPD, brain activity during free association to those sentences could have a psychodynamic “meaning”. Finally, in a recent study from our lab fMRI data is collected during free association to emotionally relevant sentences and analyzed using an OPD-based separation of subjects into two groups: (1) individuals with the association reflecting a possible psychodynamic conflict; (2) individuals who did not show any sign of conflict in their associations (Kehyayan et al., 2013). To this end, subjects’ associations were compared with the typical manifestations of the respective psychodynamic conflicts described thoroughly in the OPD manual (partnership, family, profession, etc.). The aim was to detect associations that point to probable psychodynamic conflicts regarding the theme of the stimulus sentence. OPD is thus used post-hoc to provide a psychodynamic interpretation of brain activity.

## Conclusion

In the face of profound differences between psychoanalysis and neuroscience, the critical point is to reach an intermediate level. That is, an adequate position to integrate the subjective and objective aspect of dual-aspect monism and the appropriate level to systematically investigate concepts of psychoanalysis. In this vein, the best aspects of both approaches have to be included (e.g., systematic reduction and at the same time clinically relevant expansion or reliability maintaining validity). OPD is presented as such an approach to reach that level by transferring psychodynamic constructs into neuroscience.

## Conflict of interest statement

The authors declare that the research was conducted in the absence of any commercial or financial relationships that could be construed as a potential conflict of interest.
